# Quantitative Proteomic Analysis of *Escherichia coli* Heat-Labile Toxin B Subunit (LTB) with Enterovirus 71 (EV71) Subunit VP1

**DOI:** 10.3390/ijms17091419

**Published:** 2016-08-27

**Authors:** Lin Liu, Yongping Ma, Huicong Zhou, Mingjun Wu

**Affiliations:** 1 Key Laboratory of Biochemistry and Molecular Biology, The Molecular Medicine and Cancer Research Center, Chongqing Medical University, Chongqing 400016, China; 15086785450@163.com (L.L.); zhczb@163.com (H.Z.); 2 Institute of Life Science, Chongqing Medical University, Chongqing 400016, China; wmjun20019688@163.com

**Keywords:** proteome, iTRAQ, immune, mucosal adjuvant, enterovirus 71 VP1, ETEC (Enterotoxigenic *Escherichia coli*), LTB (labile toxin B subunit)

## Abstract

The nontoxic heat-labile toxin (LT) B subunit (LTB) was used as mucosal adjuvant experimentally. However, the mechanism of LTB adjuvant was still unclear. The LTB and enterovirus 71 (EV71) VP1 subunit (EVP1) were constructed in pET32 and expressed in *E. coli* BL21, respectively. The immunogenicity of purified EVP1 and the adjuvanticity of LTB were evaluated via intranasal immunization EVP1 plus LTB in Balb/c mice. In order to elucidate the proteome change triggered by the adjuvant of LTB, the proteomic profiles of LTB, EVP1, and LTB plus EVP1 were quantitatively analyzed by iTRAQ-LC-MS/MS (isobaric tags for relative and absolute quantitation; liquid chromatography-tandem mass spectrometry) in murine macrophage RAW264.7. The proteomic data were analyzed by bioinformatics and validated by western blot analysis. The predicted protein interactions were confirmed using LTB pull-down and the LTB processing pathway was validated by confocal microscopy. The results showed that LTB significantly boosted EVP1 specific systematic and mucosal antibodies. A total of 3666 differential proteins were identified in the three groups. Pathway enrichment of proteomic data predicted that LTB upregulated the specific and dominant MAPK (mitogen-activated protein kinase) signaling pathway and the protein processing in endoplasmic reticulum (PPER) pathway, whereas LTB or EVP1 did not significantly upregulate these two signaling pathways. Confocal microscopy and LTB pull-down assays confirmed that the LTB adjuvant was endocytosed and processed through endocytosis (ENS)-lysosomal-endoplasmic reticulum (ER) system.

## 1. Introduction

*Escherichia coli* heat-labile toxin (LT) is a putative mucosal adjuvant [1,2,3,4,5,6,7,8,9]. LT is composed of one copy of the A subunit (LTA) and a homopentamer of B subunits (LTB). The LTA has ADP-ribosylation activity and the LTB has affinity for the toxin receptor of the ganglioside M1 (GM1). Both the ADP-ribosylation activity of LTA and GM1 binding of LTB are involved in immune stimulation [4,5,6,10]. However, the toxicity of LT precludes its use in humans [4,11]. LTB has been extensively studied and used as a nontoxic mucosal adjuvant experimentally [2,3,8,9,12,13]. GM1 ganglioside is the major cell surface receptor of LTB [14]. Previous studies proved that the adjuvanticity of LTB is related to GM1 binding activity [4,6,7,13]. LTB-adjuvant induces an increase in the proportion of B cells and activates most of them (CD25^+^). LTB also causes the complete depletion of CD8^+^ T-cells and increases the activation of CD4^+^ T-cells resulting in an increase in IL-2 (interleukin-2) and a decrease in IFN-γ (interferon-γ). Therefore, LTB exerts profound effects on immune cells [15]. However, a paradox report demonstrated that neither ADP-ribosylation activity nor GM1 binding are essential for adjuvanticity of LT and were therefore found to be independent of GM1-binding affinity [16]. Little is known about the mechanism of LTB adjuvanticity in host cells to date.

Enterovirus 71 (EV71) belongs to human enterovirus species A of the genus *Enterovirus* within the family *Picornaviridae*, and it contains a single open reading frame (ORF) encoding a polyprotein. The polyprotein has three genomic regions (P1, P2, and P3). The P1 encodes the capsid comprised of four structural proteins VP1, VP2, VP3, and VP4. The P2 and P3 encode the nonstructural proteins including 2A, 2B, 2C, 3A, 3B, 3C, and 3D [17]. To date, three alum-adjuvant inactivated EV71 vaccines have been developed in mainland China. These vaccines have shown high efficacy, good immunogenicity persistence, and acceptable safety profiles in clinical trials [18].

The EV71 is a major pathogen of fatal hand-foot-and-mouth disease (HFMD) in Southeast Asia and about 80% of the deaths of HFMD are caused by EV71 [17]. To date, there has only been one alum-adjuvant inactivated EV71 whole virus vaccine (FY-23K-B strain) available on the market [19]. However, even after the EV71 vaccine entry into the market, there is still a long way to go before achieving effective prevention of severe HFMD.

The VP1 subunit of EV71 (EVP1) is a neutralization antigen and induces high titers of cross-neutralizing antibodies for different EV71 subtypes, and elicits a mixed Th1 (T-helper 1 cell) and Th2 immune response with high levels of IFN-γ and IL-10. Moreover, vaccinated female mice could confer protection in their neonatal offspring [20]. Several candidate EVP1 vaccines have been reported to prevent HFMD [20,21,22,23,24,25]. Compared with the inactivated EV71, the EVP1 elicits similar humoral and cellular responses, but the engineered protein is safer, less expensive, and can be produced more efficiently [20]. However, there has not been any LTB-adjuvant EVP1 vaccine report.

In this study, we designed an LTB-adjuvant EVP1 vaccine candidate and EVP1 and LTB were constructed in pET32 and expressed in *E. coli* BL21, respectively. LTB plus EVP1 induced both humoral and mucosal immune responses via intranasal vaccination in Balb/c mice. In order to elucidate the mechanisms of the adjuvanticity of LTB, the proteomic expression profiles were detected by iTRAQ-LC-MS/MS (isobaric tags for relative and absolute quantitation; liquid chromatography-tandem mass spectrometry) in EVP1, LTB, and EVP1 plus LTB treated murine macrophage RAW264.7. The results provided a novel horizon to understand the adjuvant of LTB in the future.

## 2. Results

### 2.1. Labile Toxin B Subunit (LTB) Significantly Enhanced the Immunogenicity of Non-Replicating Enterovirus 71 VP1 Subunit (EVP1) Vaccine

**Figure 1:**
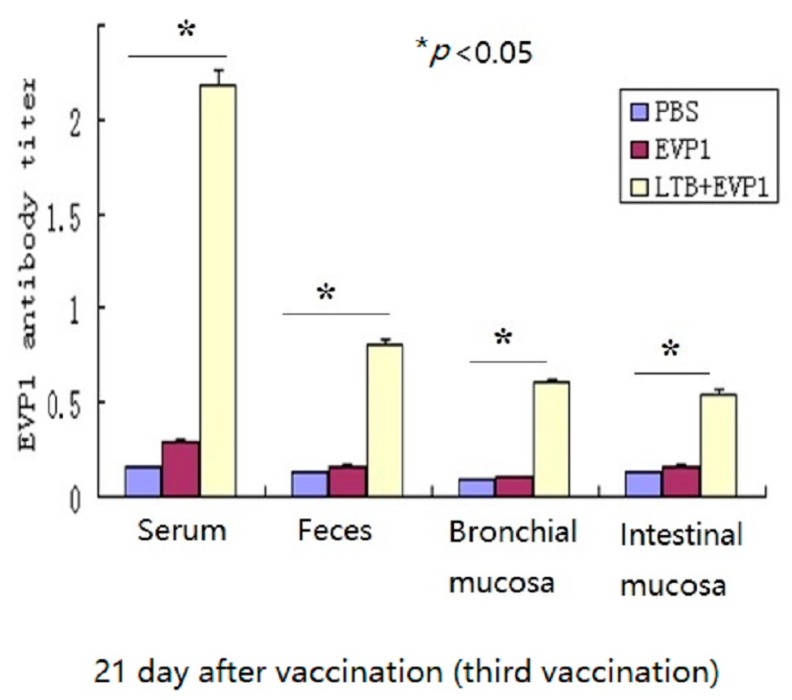
Labile toxin B subunit (LTB) significantly enhanced the immunogenicity of enterovirus 71 VP1 subunit (EVP1) vaccine. Balb/c mice of 3–4 weeks (male) were divided into four groups (6 mice in each group) as LTB, EVP1, LTB+EVP1, and PBS (phosphate buffer saline). After anesthetizing with chloral hydrate, the mice were vaccinated intranasally three times on day 0, 7, and 14 with 10–20 µL of LTB (10 µg/mL each mouse), EVP1 (10 µg/mL each mouse), LTB + EVP1 (20 µg/mL each mouse), and PBS, respectively. Samples were individually collected from immunized mice on day 21. Endpoint titers were determined as the dilution of each sample from groups of EVP1, LTB+EVP1, and PBS which showed a 2.1-fold higher absorbance level of 450 nm as compared to that of the negative control samples. Average OD_450_ values for the animals were calculated. The specific antibodies of EVP1 were significantly increased in LTB+EVP1 treatment (* *p* < 0.05).

In this study, the adjuvanticity of LTB to non-replicating EVP1 vaccine was proved via intranasal vaccination in Balb/c mice. The EVP1 specific antibody titers had no significant change between the three tested groups after 48 h of the first vaccination (*p* > 0.05, data not showed). However, both serum and mucosal EVP1 specific antibody titers were boosted significantly in group LTB plus EVP1 after 21 days of the third vaccination compared with EVP1 alone vaccination (*p* < 0.05, Figure 1). The results indicated that LTB had vigorous mucosal adjuvant activity and elicited both systematic and mucosal immune responses.

### 2.2. The Summarization of the Total Quantitative Proteome Profiles

**Figure 2:**
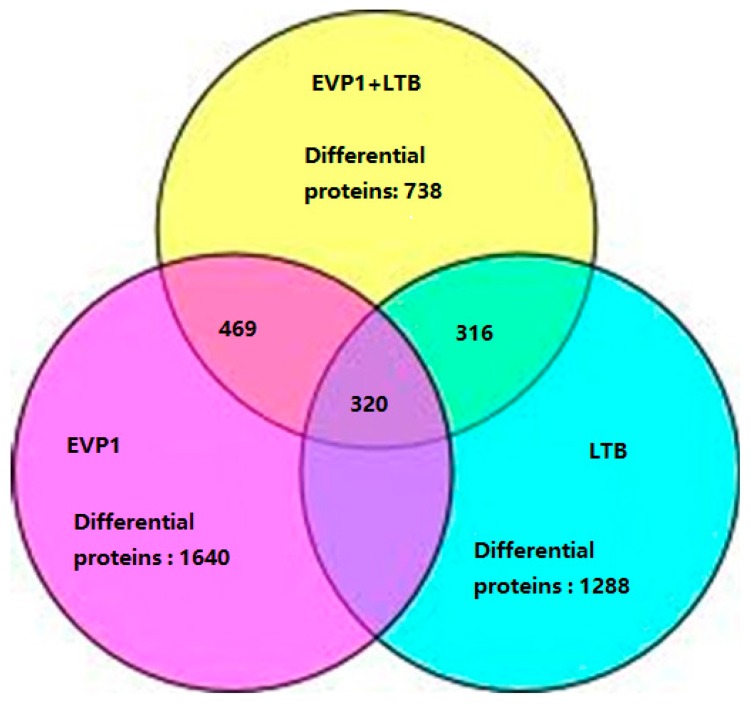
A Venn diagram to show the number of the total and overlapping differential protein profiles in different treatments. The number of total differential expression proteins and shared differential expression proteins from current work was displayed in each group. The overlapping differential proteins were obtained by comparing data from the three different groups.

A total of 4748 unique proteins were identified with 95% confidence by the ProteinPilot search algorithm against the IPI (international protein index) mouse protein database v3.49. In order to evaluate as many differential expression proteins as possible, a strict cutoff value of a ≥1.50-fold or ≤0.5-fold change resulted in a final set of 3666 differential proteins in three groups. Among of them, 1288 differential proteins were identified in group LTB (*p* ≤ 0.05); 1640 differential proteins in group EVP1 (*p* ≤ 0.05); and 738 differential proteins in group LTB plus EVP1 treatment (*p* ≤ 0.05), respectively (Figure 2). The group LTB and group EVP1 had 1040 overlapping differential proteins, the group LTB and group LTB plus EVP1 had 316 overlapping differential proteins, and the group EVP1 and group LTB plus EVP1 had 469 overlapping differential proteins. There were 320 differential proteins overlapping among the three groups (Figure 2). The results indicated that the LTB (mucosal adjuvant) and EVP1 (antigen) coaction resulted in the reduction of the total differential protein expression in mø264.7.

**Figure 3:**
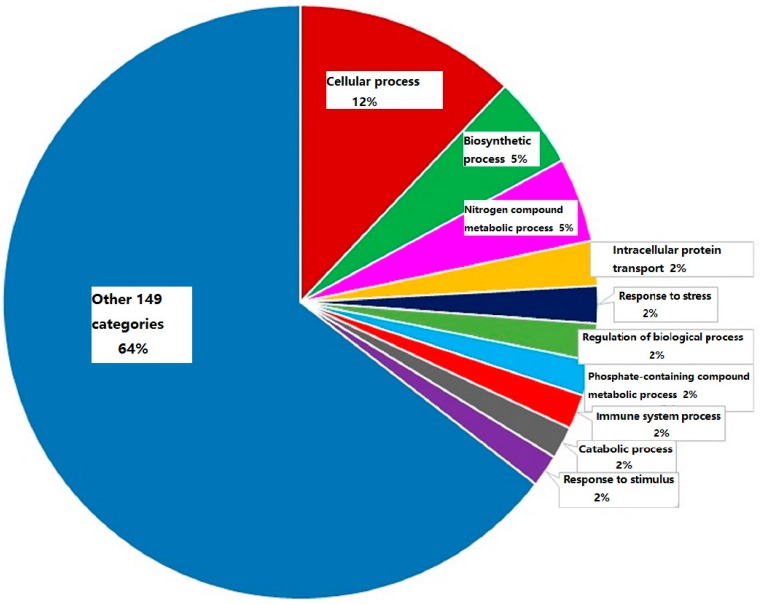
A pie chart to show the functional classification of 738 differential expression proteins in group LTB plus EVP1 treatments. The immune system processing proteins accounted for 1.9%.

A functional classification of 738 differential expression proteins in group LTB plus EVP1 was analyzed with a PANTHER tool according to the biological process with the most significance. One hundred and fifty-nine categories were observed. Among of them, the immune system processing proteins were only 1.9% in LTB plus EVP1 treatment. The category with the highest percentage of genes was cellular process (12.5%), followed by biosynthetic process (5.2%), nitrogen compound metabolic process (4.8%), intracellular protein transport (2.5%), response to stress (2.1%), regulation of biological process (2.1%), phosphate-containing compound metabolic process (2.0%), catabolic process (1.8%), and response to stimulus (1.8%), respectively (Figure 3).

### 2.3. Immune System Processing Proteins Summarization

Immune system processing differential proteins were the key issue in this study and 14 differential proteins (1.9%, 14/738) were identified according to PANTHER classification in group LTB plus EVP1. Briefly, 11 immune-associated proteins’ expression was upregulated (AHSA2, ARHGEF6, EDIL3, FCGR1, OAS1A, OASL1, SLC27A4, SLFN2, SLFN5, STAU2, and STAT1) and 3 proteins’ expression was downregulated (IFI35, PRDX1, and S100A11) (Table 1).

Similarly, 10 proteins’ expression was upregulated (A2MP, AHSA2, FCGR1, OAS1A, OASL1, OASL2, SLC11A2, SLC27A4, SLFN5, and STAT1) and five proteins’ expression was downregulated (EDIL3, GSTO1, HSPA4, PRDX1, and S100A11) (Table 1). A total of 15 immune system processing differential proteins were identified in EVP1 treatment, which accounted for 0.9% (15/1640) of the total differential proteins.

However, 13 differential proteins’ expression was increased (A2MP, AHSA2, ARHGEF6, EDIL3, FCGR1, OAS1A, OASL1, OASL2, OAS3, SLFN2, SLFN5, STAT1, and SWAP70) and 4 immune system processing proteins’ expression was decreased (PRDX1, S100AL1, GSTO1, and HSPA4) in group LTB treatment (Table 1). Therefore, at least 17 differential proteins associated with immune system processing were identified in LTB treatment mø264.7, which accounted for 1.3% (17/1280) of the total differential proteins.

As a summary, we found only three immune system processing proteins whose expression was significantly upregulated in both groups of LTB and LTB plus EVP1 without significant variation in group EVP1 (ARHGEF6, EDIL3, and SLFN2) (Table 1). The results provided novel clues about the adjuvant effect of LTB combined with soluble protein antigens such as EVP1.

### 2.4. The Dominant Pathways in Antigen Processing and Immunoregulation

To predict the dorminant pathway in the exogenous antigen processing coupled with LTB adjuvant, the pathway enrichment was processed by STRING network analysis. An important finding indicated that LTB plus EVP1 treatment upregulated the specific and dominant MAPK (mitogen-activated protein kinase) signaling pathway (1.05 × 10^−2^) and the protein processing in endoplasmic reticulum (PPER) pathway (2.11 × 10^−2^). Whereas LTB or EVP1 single treatment could not activate the MAPK or PPER signaling pathways (Table 2). The 12 upregulated MAPK signaling pathway proteins were: MAP2K3, MAP3K4, FAS, NFATC2 (nuclear factor of activated T-cells, cytoplasmic), FLNC (vascular endothelial growth factor receptor 1), NFKB1 (nuclear factor NF-κB p105 subunit), NFKB2, RAP1B (Ras-related protein Rap-1b), PPM1A (protein phosphatase 1A), CHP (calcineurin B homologous protein 1), FLNA (filamin-A), and PTPN7 (tyrosine-protein phosphatase non-receptor type 7). The results suggested that MAPK signaling pathway is a potential target for elucidating the mechanism of LTB adjuvant in future and that endoplasmic reticulum (ER) is a crucial apparatus for antigen processing and presentation.

Two other pathways were significantly upregulated in groups of LTB, EVP1, and LTB plus EVP1. The results indicated that the endocytosis (ENS) pathway and ubiquitin mediated proteolysis pathway (UBMP) were the common pathways in the three different groups (Table 2). Briefly, 13 increased expression proteins of ENS pathway were identified in LTB treatment mø264.7 (*p*-value: 4.39 × 10^−4^). The proteins were STAM2 (signal transducing adapter molecule 2), CBL (E3 ubiquitin-protein ligase CBL), DNM1L (dynamin-1-like protein), DNM2 (dynamin 2), FLT1 (vascular endothelial growth factor receptor 1), EHD1 (EH domain-containing protein 1), EHD2, SRC (proto-oncogene tyrosine-protein kinase Src), CHMP5 (charged multivesicular body protein 5), ZFYVE9 (Zinc finger FYVE domain-containing protein 9, early endosomal protein), ARFGAP1 (ADP-ribosylation factor GTPase-activating protein 1), ARFGAP2, and IQSEC1 (IQ motif and SEC7 domain-containing protein 1).

Similarly, 13 proteins of ENS pathway with increased expression were identified in EVP1 treatment (*p*-value: 7.17 × 10^−5^). The proteins were TGFB1 (transforming growth factor β-1), FLT1, DNM2, CLTA (clathrin light chain A), CBLB, EHD1, EHD2, VPS45 (vacuolar protein sorting-associated protein 45), VPS37B, VPS25, RAB4A, CHMP5, and IQSEC1. Fourteen proteins of ENS pathway with increased expression were identified in group LTB plus EVP1 (*p*-value: 4.80 × 10^−5^). The proteins were CSF1R (macrophage colony-stimulating factor 1 receptor), CBL, CBLB, EHD1, DNM1L, CLTA, CLTB, RAB4A, ZFYVE9, VPS25, CHMP5, ARFGAP2, ASAP2 (Arf-GAP with SH3 domain, ANK repeat and PH domain-containing protein 2, activates the small GTPases ARF1, ARF5, and ARF6) and IQSEC1 (Table 2).

Likewise, 10 proteins with increased expression belonging to the UBMP pathway were identified in group LTB (*p*-value: 1.17 × 10^−3^). The proteins were ANAPC2 (anaphase-promoting complex subunit 2), ANAPC4, ANAPC7, CBL, HERC4 (probable E3 ubiquitin-protein ligase HERC4), PPIL2 (peptidyl-prolyl *cis*-*trans* isomerase-like 2), UBE2R2 (ubiquitin-conjugating enzyme E2 R2), UBE2S, WWP2 (NEDD4-like E3 ubiquitin-protein ligase WWP2) and XIAP (E3 ubiquitin-protein ligase XIAP). Six proteins of the UBMP pathway with increased expression were identified in group EVP1 (*p*-value: 5.720 × 10^−6^). They were ANAPC2, ANAPC4, ANAPC7, BUB1B, CDK1 (cyclin-dependent kinase 1), and CDC16 (cell division cycle protein 16 homolog). Nine proteins of UBMP pathway with increased expression were identified in group LTB plus EVP1 (*p*-value: 2.27 × 10^−3^). They were ANAPC1, ANAPC4, ANAPC7, CBL, CBLB, CDC16, KLHL9 (kelch-like protein 9, substrate-specific adapter of a BCR (BTB-CUL3-RBX1) E3 ubiquitin-protein ligase complex), UBE2R2, and UBE2S. The results suggested that these two pathways contained large numbers of upregulated expression genes. The data implied that receptor-mediated ENS-UBMP pathways might play critical roles in LTB, EVP1, and LTB plus EVP1 antigen processing in mø264.7 (Table 2).

### 2.5. Confocal Microscopy Assay Indicated That LTB Was Endocytosed in ENS-Lysosomal-ER System

**Figure 4:**
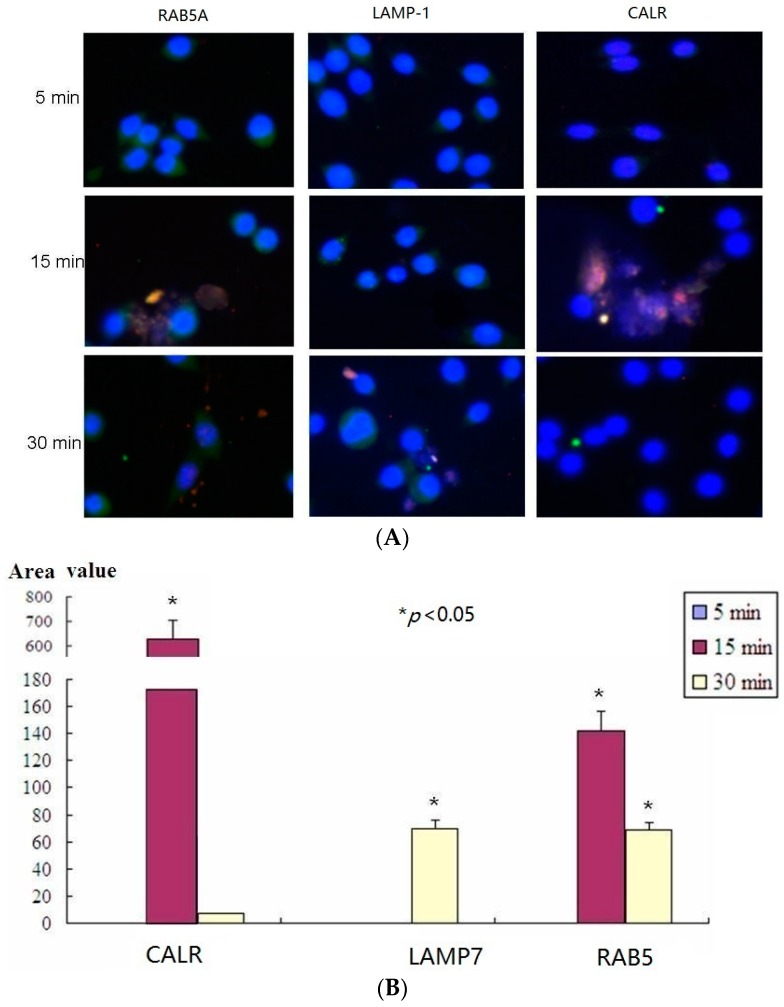
Results of confocal microscopy assay. (**A**) mø264.7 cells were treated with LTB and fixed at 5, 15, or 30 min, respectively. After penetration of 0.5% Triton-X 100, the samples were blocked with 10% BSA/PBS and incubated with the rabbit-anti-RAB5A, rabbit-anti-LAMP-1, rabbit-anti-CALR, and mouse-anti-His-tag. Then the samples were incubated with secondary DyLight 488 labeled goat anti-rabbit IgG and Cy3 labeled goat anti-mouse IgG to detect the RAB5A, LAMP-1, CALR (**green**) and LTB (**red**), respectively. Images were taken using a confocal laser scanning microscope (FluoView1000, Olympus, Tokyo, Japan) with a 20× objective using the sequential scanning mode (200×) and processed using the FluoView software (Olympus); and (**B**) Quantitative analysis of microscopy images (* *p* < 0.05).

**Figure 5:**
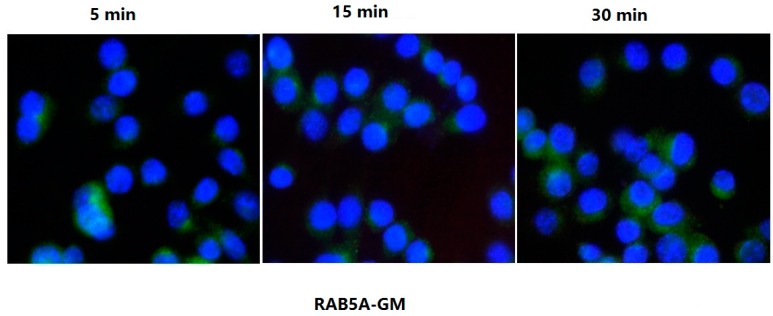
Results of the internalization of LTB inhibition. mø264.7 cells were treated with 100 nM ganglioside M1 (GM) for 5, 15, or 30 min, respectively. The LTB was added and incubated for another 5, 15, or 30 min, respectively. After fixing in a solution of 4% formaldehyde and penetrating with 0.5% Triton-X100, the samples were blocked with 10% BSA/PBS and incubated with the rabbit-anti-RAB5A and mouse-anti-His-tag of LTB. Then the samples were incubated with secondary DyLight 488 labeled goat anti-rabbit IgG and Cy3 labeled goat anti-mouse IgG to detect the RAB5A (**green**) and LTB (**red**), respectively. Images were taken using a confocal laser scanning microscope (FluoView1000, Olympus) with a 20× objective using the sequential scanning mode (200×) and processed using the FluoView software (Olympus).

To evaluate LTB binding and entry in live mø264.7 cell through surface GM1, several molecular markers were examined by confocal microscopy at different time points. Figure 4 and Figure 5 confirm the results that LTB entered through GM1 binding and was processed via ENS pathway (Figure 4 and Figure 5). The results showed that RAB5A colocalized with LTB at 15 and 30 min (Figure 4). RAB5A was required for the fusion of plasma membranes and early endosomes during exogenous antigen processing. Therefore, it confirmed that LTB was processed through ENS pathway. However, CALR was observed to interact with LTB in ER at 15 min. There was no CALR-LTB colocalization signal detectable at 30 min in ER (Figure 4). Sequently, LAMP-1 was observed to colocalize with LTB in lysosome at 30 min only (Figure 4). The results suggested that LTB was endocytosed in ENS-lysosomal-ER system and that the process might last for 30 min.

The results of LTB inhibition analysis showed that LTB did not colocalize with RAB5A in early endosomes from 5 to 30 min after being treated with soluble GM (ganglioside M1), a typical LT inhibitor (Figure 5). Therefore, the endocytosis of LTB was completely inhibited by GM.

### 2.6. His-LTB Pull-Down Assay

To detect the interaction of LTB with target proteins of mø264.7, LTB pull-down assay was performed. Seventy proteins were pulled down with His-LTB. Among of them, 13 proteins were upregulated more than 1.5-fold in LTB treatment, and 16 proteins were downregulated more than 0.5-fold. However, the other 41 proteins showed no significant variation (Table S1). In order to predict the validity of protein-protein interaction of the LTB pull-down proteins, the proteins were processed by STRING network analysis. The results showed that 60 proteins interacted with each other except for 10 proteins (RAB1, UBAP2l, PRSS1 (protease serine 1), CFL1 (cofilin-1), AHCY (adenosylhomocysteinase), ALDH2 (aldehyde dehydrogenase, mitochondrial), ACAA1A (3-ketoacyl-CoA thiolase A, peroxisomal), CTSB (cathepsin B), FDPS (farnesyl pyrophosphate synthase) and HIST1H1E (histone H1E)) (Figure S1). This information suggested that most of the proteins might be pulled down indirectly by LTB. For instance, a TCP1 (t-complex protein 1) protein and six CCT (chaperonin containing TCP1) proteins were pulled down by LTB. However, their role in the folding of actin and tubulin was established.

### 2.7. The Western Blot Confirmed the Validity of iTRAQ-LC-MS/MS Data

**Figure 6:**
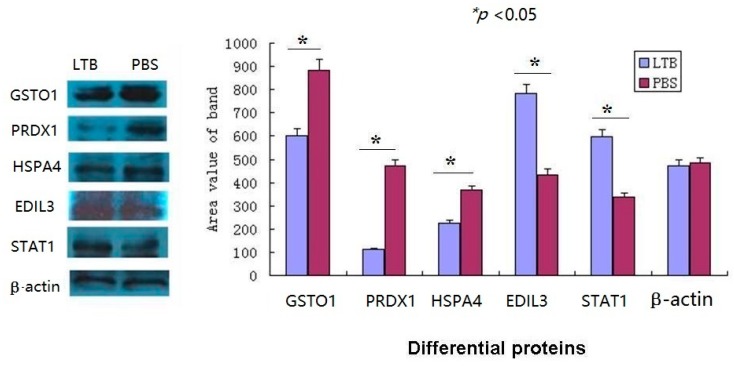
Western blot analysis of some differential proteins. The target proteins of LTB treated mouse mø264.7 was expressed in line with the trend of iTRAQ-MS analysis results. The area value of protein bands was measured by Image-pro plus tools and the proteins change significantly (* *p* < 0.05). More than three biological replicates were performed.

To test the validity of iTRAQ-LC-MS/MS data, the western blot was performed and the results proved that the expression of the differential proteins (EDIL3, STAT1, HSPA4, GSTO1, PRDX1) was consistent with the data obtained from iTRAQ (repeats > 3, Figure 6). These proteins were classified as immune system process proteins by PANTHER. The results validated the compounds identified in Table 1.

## 3. Discussion

Mucosal immunization requires adjuvant and efficient carrying vehicles as delivery systems [6]. In recent years, designing safe and effective mucosal vaccines and adjuvants has been a challenge [11]. Only several vaccines are routinely administered mucosally to humans (e.g., poliomyelitis, *Vibrio cholerae*, *Salmonella typhi*, rotavirus, and influenza).

Even though LTB is a nontoxic mucosal adjuvant and widely used experimentally [2,3,8,9,12,13], the use of LTB will not overcome the major limitation of using LT or mutants of LT intranasally in humans [16]. All of these molecules still traffic back to the brain through the olfactory neurons as a function of GM1 binding [16,26]. So LTB is not safe for intranasal use in humans. Other evidence suggested that intracerebral injections of LTB at doses less than 22.7 μg/mouse does not cause any significant adverse effects on the brain in animal models [11]. LTB can therefore be used as adjuvant via non-intranasal route, e.g., via oral vaccination [8,9,10].

HFMD is caused by intestinal viruses of the *Picornaviridae* family, such as EV71, and has become a serious public health problem in southeast Asia [27]. Mucosal surfaces are the major entrance for HFMD pathogens and therefore mucosal immune responses serve as a first line of defense. This study was designed as a non-replicating mucosal vaccine of EVP1 for HFMD prevention.

As a summary, most of the immune system processing differential proteins identified in this study were directly involved in immune responses. For instance, A2MP was able to bind endogenous or foreign peptides, providing a barrier against pathogens, and inhibit all four classes of proteinases by a unique “trapping” mechanism, leading to clearance [28]. Moreover, A2MP also bound cytokines and growth factors by another mechanism of stabilization of biologically active molecules, preventing clearance [28]. AHSA2 acted as a cochaperone that stimulated HSP90 ATPase activity. Then the HSP90-peptide complexes stimulated antigen presentation through the class II pathway [29]. ARHGEF6 was a RAC1 guanine nucleotide exchange factor (GEF). Mice lacking ARHGEF6 had reduced numbers of mature lymphocytes and defective immune responses [30]. EDIL3 promoted adhesion of endothelial cells through interaction with the α-v/β-3 integrin receptor and was an important endogenous inhibitor of inflammatory cell adhesion and homing [31]. FCGR1 (CD64) functioned in both innate and adaptive immune responses. Antigens bound to FCGR1 via antibody could be shunted to the class I as well as the class II pathways in monocytes and efficiently presented to CD8 or CD4 cells, respectively [32,33]. HSPA4 was involved in the antigen presentation and cross-presentation for specific triggering of the acquired immune response [34]. IFI35 served as an interferon-induced protein and negatively regulated RIG-I antiviral signaling [35]. 2′–5′ oligoadenylate synthetases (OAS1A, OASL1, OASL2, and OAS3) played a role in broad antiviral activity mediated through RNase L, which was activated by IFN-α [36,37]. S100A11 facilitated the differentiation and the cornification of keratinocytes. Inhibition of S100A11 gene expression impaired the ability of keratinocytes to control viral vaccine replication via downregulation of IFN-λ receptor IL-10R2 [38]. Solute carrier 11 (SLC11) or natural resistance-associated macrophage protein (NRAMP) were proton-coupled transporters that facilitated absorption of divalent metal ions (Fe^2+^, Mn^2+^, Zn^2+^). It was reported that SLC11A1 limited intracellular growth of *Salmonella enterica* sv. Typhimurium by promoting macrophage immune effector functions and impairing bacterial iron acquisition [39]. Schlafens (SLFN2, SLFN5) were a group of IFN-inducible proteins involved in the control of cell cycle progression and growth inhibitory responses. They might have a role in hematopoeitic cell differentiation and induction of immune responses. For instance, SLFN2 had been implicated in a variety of functions, such as contributing to an immune response, differentiation, and to cell growth [40,41]. SLFN4 mRNA levels were upregulated during macrophage activation but downregulated during differentiation [42]. STAT1 was a transcription factor that bound to the IFN-stimulated response element (ISRE). STAT1 signaling was essential for regulation of immune polarization and activation of macrophages which occurred during protective anti-pathogen immune responses [43]. SWAP70 restricted spontaneous maturation of dendritic cells and controlled surface localization of MHC (major histocompatibility complex) II, and was required for efficient B cell homing to lymphoid organs [44,45].

On the other hand, several proteins regulated immune responses by reactive oxygen species (ROS) regulation pathway. It was confirmed that ROS were involved in the regulation of T-cell mediated physiological and pathological processes and played an important role in T-cell activation [46]. After the recognition of bacteria by the TLRs (toll-like receptor) in macrophages, the signaling cascade led to the generation of ROS (oxidative burst), the oxidative burst was bactericidal by causing lipid, protein, and DNA lesions, resulting in pathogen clearing [47]. As reductases, GSTO1 and PRDX1 were involved in ROS regulation. For instance, knockdown of GSTO1-1 in macrophage-like cells blocked the expression of NADPH oxidase 1 and the generation of ROS after LPS (lipopolysaccharide) stimulation [48]. GSTO1-1 is required for LPS-mediated signaling in macrophages and as it acts early in the LPS-TLR4 pro-inflammatory pathway [49]. STAU2 served as a RNA-binding protein and regulated mRNA localization, mRNA stability, translation, and ribonucleoprotein (RNP) assembly [50]. However, there is no evidence to prove the involvement of STAU2 in immune responses to date.

The exogenous antigen proteins may be endocytosed and degraded within the cell [51,52]. The major role of the endosomal system was to process the internalization of antigen from the cell surface into the endosomal system and dynamically trafficking antigen molecules between intracellular compartments and the cell surface, which regulated both innate and adaptive immune responses [53].

The ubiquitin-proteasome system plays an important role in various cellular processes. Ubiquitination requires E1 and either of two types of E2s and E3s. Most of the ENS-UBMP pathway proteins were ubiquitin ligases. For instance, UBE2R2 and UBE2S were E2 enzymes and acted via selective protein-protein interactions with the E1 and E3 enzymes and connected activation to covalent modification [54]. ANAPC served as multisubunit E3 ubiquitin ligases that initiated chromosome segregation and mitotic exit by targeting critical cell-cycle regulators for proteolytic destruction [55]. ARFGAP regulated vesicular traffic and actin cytoskeleton dynamics in mammalian cells [56]. ASAP2 was involved in the regulation of vesicular transport, cellular migration, and autophagy [57]. CBL and CBLB were tyrosine kinase-directed RING finger type E3 ubiquitin ligases [58,59]. CBLB also regulated innate immune responses and played an important role in host defense to pathogens [58,60]. The knockout of CBLB led to increased adaptive and innate antitumor immunity [59]. RAB GTPase activating proteins (RABGAPs) containing TBC (TRE2-BUB2-CDC16) domain were important factors for the coordination of cellular vesicle transport systems [61]. HERC4 played a role of E3 ubiquitin ligase and immunofluorescence showed HERC4 localization to the endosome and lysosomes [62]. RAB4A was a small GTPase and a master regulator of receptor recycling from endocytic compartments to the plasma membrane [63]. WWP2 was a ubiquitin E3 ligase and proved to degrade a series of targets via a ubiquitin-dependent proteasome system [64,65].

The other ENS-UBMP pathway proteins belonged to endocytosis proteins. For instance, DNM (dynamin) was essential for membrane fission during clathrin-mediated endocytosis in eukaryotic cells. T-cell-specific deletion of DNM2 resulted in reducing TCR signaling strength [66]. CHMP5 regulated late endosome function downstream of multivesicular body formation, and the loss of CHMP5 enhanced signal transduction by inhibiting lysosomal degradation of activated receptors [67]. EHD was an endocytic recycling protein and involved in intracellular trafficking, especially endocytic recycling [68]. Clathrin served as a molecular scaffold for vesicular uptake of cargo at the plasma membrane and clathrin-mediated endocytosis (CME) was one of the main methods of cellular uptake [69,70]. IQSEC1 (GEP100, BRAG2) selectively activated ARF6 (ADP ribosylation factor 6) during integrin internalization and ARF6 regulated both endocytosis and recycling of β1 integrins [71]. VPS, classified into class A to F, was involved in vesicle trafficking and was required for the sorting of ubiquitinated transmembrane proteins into internal vesicles of multivesicular bodies [72,73].

The confocal microscopy assay is a useful method to visualize antigen processing and protein-protein interaction or colocalization [74,75]. Our data showed the evidence of exogenous LTB processing in endosomal system in chronological order, visually. The confocal microscopy showed that RAB5A, similar to RAB1, interacted with LTB confirming the validity of LTB pull-down assay. Therefore, both confocal microscopy and LTB pull-down assay indicated that LTB was processed via endocytosis in endosomal-lysosomal-ER system by GM1 receptor mediating.

GM1 ganglioside is the major cell surface receptor of LTB [16]. Gangliosides are major components of lipid rafts [76]. The first step of T-cell activation involves movement of the T-cell receptor (TCR) into lipid rafts. The different types of T-cells require distinct ganglioside types for the activation. CD4 T-cells require a-series gangliosides for activation. CD8 T-cells require 0-series gangliosides for activation [76]. That may explain why GM1-binding deficient mutation LTB (G33D) lost its mucosal adjuvant [15,77].

Macrophages, even though they are not equal to DC (dendritic cell), have been used as APC (antigen-presenting cell) models in previous studies [78,79,80]. Therefore, the proteomic profiles obtained in mø264.7 provided useful information of LTB adjuvanticity [81]. No cytokines were detected from mø264.7 cell lysates. However, cytokines play important roles in the innate and adaptive immunity and appear to interact functionally in networks [82]. It was reported that mutant *E. coli* LT (R192G/L211A) enhanced IL-17A production in human T-cells specific for bacterial vaccine antigens [5]. The IL-17A enhancing effect of mutant LT was suppressed by neutralization of IL-1β and IL-23, but not IL-6 [5]. Therefore, the cytokine profiles will be analyzed with purifying dendritic cells or T-cells supernatant from differentially immunized animals in future.

This study evaluated the differential proteomic profiles treated by non-replicating mucosal EVP1 subunit vaccine plus LTB adjuvant using proteomics method in a mø264.7 model. The data demonstrates that LTB was processed via endocytosis in endosomal-lysosomal-ER system. To our knowledge, it was the first study to suggest that LTB plus EVP1 upregulated the specific and dominant MAPK and PPER signaling pathways, whereas LTB or EVP1 did not activate the dominant MAPK or PPER signaling pathway. That meant that MAPK and PPER signaling pathways played important roles in LTB-adjuvant vaccine immune responses.

ER is a subcellular organelle where proteins are folded with the help of lumenal chaperones. Newly synthesized peptides enter the ER via the sec61 pore and are glycosylated. Correctly folded proteins are packaged into transport vesicles that are shuttled to the Golgi complex. Our immunofluorescence microscopy assay indicated that LTB was also processed in ER for its colocalization with CALR.

MAPKs are serine and threonine protein kinases [83]. MAPK signaling pathway is a highly conserved module that is involved in various cellular functions, including cell proliferation, differentiation, and migration. The immune response is one of several key functions regulated by MAPKs, with the production of immunomodulatory cytokines, such as TNFα, IL-1, IL-10, and IL-12, induced by the activation of p38 MAPK, JNK (c-Jun N-terminal kinase), and ERK (extracellular signal-regulated kinas) pathways [83,84].

Next in the study, we purified bone-marrow derived dendritic cells from LTB, EVP1, and LTB plus EVP1 immunized mice and studied the LTB adjuvant mechanism under the guidance of the specific and dominant MAPK signaling pathway associated upregulation proteins` enrichment in a mø264.7 model.

## 4. Materials and Methods

### 4.1. EVP1 and LTB Purification

Full-length of DNA encoding EVP1 (GenBank: AB204852.1) was commercially synthesized (Sangon, Shanghai, China). Full-length LTB DNA was cloned from EC44815 strain (national institutes for food and drug control, NIFDC) as described previously [8]. The *ltb* and *evp1* DNAs were constructed into plasmid pET32 at *Bam*H I/*Sal* I site, respectively. The recombinant LTB and EVP1 were expressed in *E. coli* BL21 cells and purified with BeaverBeads™ His-tag protein purification kits (Beaver, Suzhou, China). The endotoxin was removed using ToxinEraser^TM^ resin (Genscript, Nanjing, China) and the protein concentration was measured by the BCA protein assay kit (Genscript).

### 4.2. Animal and Immunization

Balb/c mice of 3–4 weeks (male) were bred in the experimental animal center (Chongqing Medical University, Chongqing, China) and divided into four groups. Six mice were included in each group that is, LTB, EVP1, LTB + EVP1, and PBS. After anesthetizing with chloral hydrate (0.5 mL/100 g), the mice were intranasally vaccinated three times on day 0, 7, and 14 with 10–20 µL of LTB (10 µg/mouse), EVP1 (10 µg/mouse), LTB+EVP1 (20 µg/mouse), and PBS. This study was carried out in strict accordance with the recommendations in the Guide for the Care and Use of Laboratory Animals of the National Institutes of Health. The protocol was approved by the Committee on the Ethics of Animal Experiments at the Chongqing Medical University (SYXK2012-0001, 2013-03-11). All surgery was performed under sodium pentobarbital anesthesia, and euthanized by cervical dislocation.

### 4.3. Immunological Assay

Blood samples were individually collected from immunized mice by tail bleeding on days 0, 7, 14, and 21 for the analysis of systemic EVP1-specific antibodies (6 mice in each group). Fresh fecal pellets were individually collected and lyophilized from the same mice groups on days 0, 7, 14, and 21. The samples of small intestine mucosal washing and bronchial mucosal washing were washed from euthanized mice on day 21 after third vaccination (6 mice each group). All the samples were treated as previously described [8]. The supernatants of fecal washing, small intestine mucosal washing and bronchial mucosal washing were analyzed for EVP1-specific sIgA to evaluate the mucosal immune response. The samples collected on 48 h post-vaccination and 21 day (7 day post-third-vaccination) were analyzed with horseradish peroxidase (HRP)-labeled goat anti-mice IgG and goat anti-mice IgA (1.0 μg/mL, Boster, Wuhan, China) by ELISA, respectively, as previously described [21].

Endpoint titers were determined as the dilution of each sample showing a 2.1-fold higher absorbance level of 450 nm as compared to that of the negative control samples. Average OD_450_ values for the animals were calculated. EVP1 specific antibody were not detected in the LTB mice group due to its variation which is inconsistent with PBS treatment.

### 4.4. Cells, Cell Culture, and Cell Treatment

Macrophage 264.7 (mø264.7) was purchased from American type culture collection (ATCC, Manassas, VA, USA) and maintained in RPMI 1640 complete growth medium. The cells were cultured in 250 cm^2^ flasks and grown to 70% confluence in RPMI 1640 medium at 37 °C and 5% CO_2_. Then the cells were treated with LTB (100 μg/flask), EVP1 (100 μg/flask), and LTB (100 μg/flask) plus EVP1 (100 μg/flask) for 24 h. Equal volume PBS (endotoxin free) was added to another flask of cells as a negative control.

### 4.5. Cell Sample Preparation

The cell cultures were centrifuged at 2000× *g* for 30 min at 4 °C and the cells were lysed in lysis buffer (7 M urea, 1 mg/mL DNase I, 1 mM Na_3_VO_4_, and 1.0 μL protease inhibitor cocktail (Sigma P8340, Sigma-Aldrich, Shanghai, China). The cell lysates were centrifuged at 4000× *g* for 30 min at 4 °C, respectively. Then the supernatants of cell lysates were collected for iTRAQ labeling or for western blot assay. The protein concentrations were measured by the BCA protein assay reagent kit (Pierce, Appleton, WI, USA).

### 4.6. Digestion and iTRAQ Labeling

Each 100 μg of whole cell proteins from PBS, LTB, EVP1, and LTB plus EVP1 treated cells were digested with 20 μL trypsin solution (0.1 μg/μL, Promega, Madison, WI, USA) at 37 °C overnight and then labeled with the iTRAQ tags (PBS group, 114 tags; LTB group, 115 tags; EVP1 group, 116 tags; LTB plus EVP1 group, 117 tags). After 2 h, labeled peptides were combined and purified using cation-exchange and C18 cartridges. Briefly, (a) we diluted the samples 10-fold in SCX (strong cation exchange) buffer A and added in a 2.1 × 200 mM polysulfoethyl A SCX column (PolyLC; Columbia, MD, USA); (b) Then we eluted the column with a gradient of 0%–25% SCX buffer B over 30 min, followed by a gradient of 25%–100% SCX buffer B over 40 min; (c) We collected the fractions at 1 min intervals and lyophilized it in a vacuum concentrator and desalted it using a C18 reverse-phase column (Discovery DSC-18 SPE, Supelco; Sigma-Aldrich, Shanghai, China); (d) Finally, we lyophilized the desalted fractions again and stored them at −20 °C for mass spectrometric (MS) analysis [85].

### 4.7. Mass Spectrometric Analysis

MS was performed using a nano-LC coupled online to a QStar Elite mass spectrometer (Applied Biosystems, Waltham, MA, USA). Peptides were loaded on a fused silica C18 reverse-phase capillary column (75 µm id × 10 cm), followed by a mobile phase elution with buffer A (0.1% formic acid in 2% acetonitrile) and buffer B (0.1% formic acid in 98% acetonitrile). The peptides were eluted from 2% buffer B to 100% buffer B over 60 min at a flow rate of 300 nL/min. The LC eluent was directed to an electrospray ion (ESI) source for quadrupole time-of-flight mass spectrometry (Q-TOF-MS) analysis. The mass spectrometer was set to perform information-dependent acquisition (IDA) in the positive ion mode (mass range of 300–2000 *m*/*z*). Peptides with +2 to +4 charge states were selected for MS/MS, and the time of summation of MS/MS events was set to 3 s. The two most abundantly charged peptides above a 10-count threshold were selected for MS/MS and dynamically excluded for 60 s with ±50 mmu mass tolerance.

Peptide identification and quantification were performed using ProteinPilot software packages (Applied Biosystems). Each MS/MS spectrum was searched against the international protein index (IPI) mouse protein database, and protein identification was accepted depending on ProteinPilot confidence scores. Error factor and *p*-value were calculated using ProteinPilot software, which gave an indication of the deviation and significance in the protein quantification (*p* ≤ 0.05 for statistical significance).

### 4.8. Data Analysis

To determine the possible immunological consequences after LTB, EVP1, and LTB plus EVP1 stimulation in mø264.7, the down- and upregulated proteins were classified using the PANTHER classification system v9.0 (PANTHER, protein annotation through evolutionary relationship, http://www.pantherdb.org) [86]. The immune-associated differential proteins were identified from the different administrations. The antigen processing proteins were predicted using pathway enrichment of STRING v9.1 (http://string-db.org) [87].

### 4.9. Confocal Microscopy Assay

The rabbit IgG1-mAbs used in confocal microscopy were anti-RAB5A (Ras-related protein Rab-5A; RAB5A was required for the fusion of plasma membranes and early endosomes), anti-LAMP-1 (Lysosome-associated membrane glycoprotein 1; this protein shuttled between lysosomes, endosomes, and the plasma membrane) and anti-CALR (Calreticulin, which was located in storage compartments associated with the endoplasmic reticulum (ER)). mø264.7 cells were seeded onto coverslips for 12–16 h and treated with LTB (10 µg/flask) for 5, 15, and 30 min in RPMI 1640 complete growth medium. Then cells were fixed in a solution of 4% formaldehyde (Boster) at room temperature (RT) for 20 min. After washing three times with PBS, the cells were penetrated with solution of PBS containing 0.5% Triton-X 100 for 10 min and then were blocked in 10% BSA/PBS for 1 h at 37 °C. Cells were incubated with the rabbit-anti-RAB5A, rabbit-anti-LAMP-1, rabbit-anti-CALR, or mouse-anti-His-tag for 2 h at 37 °C. Then Cells were washed five times in PBS and incubated with secondary DyLight 488 labeled goat anti-rabbit IgG (Boster) or Cy3 labeled goat anti-mouse IgG for 1 h in darkness. Finally, the cells were stained with DAPI (4′,6-diamidino-2-phenylindole) for 15 min in darkness and washed five times before blocking with 70% glycerol. Images were taken using a confocal laser scanning microscope (FluoView1000, Olympus) with a 20× objective using the sequential scanning mode.

The inhibition of LTB internalization in mø264.7 was performed with 100 nM GM1 treatment for 5, 15, and 30 min in RPMI 1640 complete growth medium. Then the internalization of LTB and the colocalization of LTB and RAB5A were detected respectively, as previously described. All images were collected and processed using the FluoView software (Olympus) and Image-Pro plus.

### 4.10. His-Flag Pull-Down Assay

In order to analyze the proteins that interacted with LTB, His-LTB was expressed and purified from *E. coli* BL21 cells as described previously. His-LTB was then run on a 5%–10% SDS-PAGE and stained with coomassie blue to analyze the concentration of the protein. Two-fold concentrations of soluble His-LTB were absorbed by 1 ml nickel magnetic beads (BeaverBeads™, Beaver, Suzhou, China) for 30 min at RT. Then the His-LTB beads were washed three times with washing buffer for 5 min each time. The His-LTB was finally eluted with elution buffer and the His-LTB collection was refolded and used again for pull-down assay.

The purified His-LTB (200 µg) was incubated with 2 mL whole mø264.7 cell lysates at 4 °C overnight. Then the pull-down samples were absorbed again and purified with BeaverBeads™ (Beaver) as previously described. Equal volumes of nickel magnetic beads and the lysates of whole mø264.7 cell were also used as negative-control. The pull-down proteins were eluted with elution buffer. The samples were digested with trypsin solution (100 μg samples containing 2.0 μg trypsin, Promega) at 37 °C overnight for LC-MS/MS assay as described previously.

The pull-down proteins were analyzed using STRING v9.1 to predict the validity of protein-protein interactions with a high confidence score (0.4) and Kmeans clustering [87].

### 4.11. Western Blot Assay

The target proteins were detected with the antibodies of EDIL3 (EGF-like repeat and discoidin I-like domain-containing protein 3; cat# 12580-1-AP), STAT1 (signal transducer and activator of transcription 1, cat# 10144-2-AP), PRDX1 (peroxiredoxin-1, cat# 15816-1-AP), HSPA4 (heat shock 70 kDa protein 4, cat#21206-1-AP), GSTO1 (glutathione *S*-transferase ω-1, cat# 15124-1-AP), and β-Actin (14395-1-AP) (Proteintech, Wuhan, China), followed by incubation with a secondary antibody conjugated with HRP. These western blots were carried out in at least three repeats. The accurate densitometry analysis was carried out using Image-Pro plus.

### 4.12. Statistical Analysis

The data was statistically evaluated by the SPSS 19.0 statistical software package (SPSS Inc., Chicago, IL, USA) with one-way ANOVA and a value of *p* < 0.05 was considered significant.

## Figures and Tables

**Table 1:** Immune system processing related differential proteins (21proteins).

Protein (Acc.#)	LTB	EVP1	LTB + EVP1	Functions
A2MP (Q6GQT1)	12.2462	2.1478	**0.6607**	It is able to bind endogenous or foreign peptides, providing a barrier against pathogens.
* ARHGEF6 (Q8K4I3)	1.6904	**1.2474**	1.6904	Acts as a RAC1 guanine nucleotide exchange factor (GEF) and positive regulates immune responses.
AHSA2 (Q8N9S3)	2.1677	2.1281	1.5704	Cochaperone that stimulates HSP90 ATPase activity and stimulates antigen presentation through the class II pathway.
* EDIL3 (O35474)	2.2699	0.4742	1.7219	An important endogenous inhibitor of inflammatory cell adhesion and homing.
FCGR1 (P26151)	2.0512	3.1046	2.1086	Functions in both innate and adaptive immune responses.
GSTO1 (O09131)	0.3981	0.2858	**0.6427**	Exhibits glutathione-dependent thiol transferase and dehydroascorbate reductase activities.
HSPA4 (Q3U2G2)	0.4487	0.1754	**0.7178**	Involved in the antigen presentation and cross-presentation for specific triggering of the acquired immune response.
IFI35 (Q9D8C4)	1.3932	0.6081	0.4246	Interferon-induced protein 35 and negatively regulated RIG-I antiviral signaling.
OASL1 (Q8VI94)	3.3113	3.1623	2.0701	May play a role in mediating resistance to virus infection, control of cell growth, differentiation, and apoptosis.
OASL2 (Q9Z2F2)	2.466	2.3768	**1.4322**	May play a role in mediating resistance to virus infection, control of cell growth, differentiation, and apoptosis.
OAS1A (P11928)	1.977	1.6293	1.556	May play a role in mediating resistance to virus infection, control of cell growth, differentiation, and apoptosis.
OAS3 (Q8VI93)	1.7061	**1.3305**	**1.2023**	May play a role in mediating resistance to virus infection, control of cell growth, differentiation, and apoptosis.
PRDX1 (P35700)	0.1888	0.0738	0.4699	Reduces peroxides with reducing equivalents provided through the thioredoxin system but not from glutaredoxin.
S100A11 (P50543)	0.1445	0.0649	0.2228	Facilitates the differentiation and the cornification of keratinocytes and resists to virus infection.
* SLFN2 (Q9Z0I6)	2.208	**1.2023**	1.6144	May have a role in hematopoeitic cell differentiation, induction of immune responses.
SLFN5 (Q8CBA2)	7.8705	6.6681	3.8371	May have a role in hematopoeitic cell differentiation, induction of immune responses.
SLC11A2 (P49282)	**1.3183**	2.421	**1.4322**	May play an important role in hepatic iron accumulation and tissue iron distribution.
SLC27A4 (Q91VE0)	**1.1695**	1.977	1.5704	Plays a role in the formation of the epidermal barrier. Required for fat absorption in early embryogenesis.
STAU2 (Q8CJ67)	**1.0864**	**1.4723**	1.8535	RNA-binding protein, mRNA stability, translation.
STAT1 (Q8C3V4)	2.466	2.2284	1.8197	Transcription factor that binds to the IFN-stimulated response element (ISRE) and to the GAS element.
SWAP70 (Q6A028)	1.5996	**0.8872**	**1.3428**	Restricts spontaneous maturation of dendritic cells.

0.5001–1.4999 means no variation and marked with boldface. The stars (*) marked the differential proteins expression which was significantly upregulated in both groups of LTB and LTB plus EVP1 without significant variation in group EVP1.

**Table 2:** Summarization of pathway enrichment.

GO_ID	Pathway	EVP1	LTB	LTB + EVP1
		*p*-Value	*p*-Value	*p*-Value
mmu04144	Endocytosis	* 7.17 × 10^−5^	* 4.39 × 10^−^^4^	* 4.80 × 10^−5^
mmu04120	Ubiquitin mediated proteolysis	* 5.72 × 10^−^^6^	* 1.17 × 10^−^^3^	* 2.27 × 10^−^^3^
mmu04141	Protein processing in endoplasmic reticulum	2.02 × 10^−^^1^	7.21 × 10^−^^1^	* 2.11 × 10^−2^
mmu04010	MAPK signaling pathway	6.26 × 10^−^^1^	4.07 × 10^−^^1^	* 1.05 × 10^−2^
mmu04612	Antigen processing and presentation	* 3.21 × 10^−^^2^	2.34 × 10^−1^	1.00 × 10^0^

* Significant signaling pathway (* *p* < 0.05).

## Supplementary Materials

Supplementary materials can be found at www.mdpi.com/1422-0067/17/9/1419/s1.

## Abbreviations

A2MP	α-2-macroglobulin-P
ACAA1A	3-ketoacyl-CoA thiolase A
AHCY	adenosylhomocysteinase
AHSA2	activator of 90 kDa heat shock protein ATPase homolog 2
AKT3	RAC-α serine/threonine-protein kinase 3
ALDH2	aldehyde dehydrogenase, mitochondrial
ANAPC2	anaphase-promoting complex subunit 2
ARF6	ADP ribosylation factor 6
ARFGAP	ADP-ribosylation factor GTPase-activating protein
ARHGEF6	ρ guanine nucleotide exchange factor 6
ASAP2	Arf-GAP with SH3 domain, ANK repeat and PH domain-containing protein 2
CALR	Calreticulin
CBL	E3 ubiquitin-protein ligase CBL
CBLB	E3 ubiquitin-protein ligase CBL B
CCND1	G1/S-specific cyclin-D1
CCT	chaperonin containing TCP1
CDK1	cyclin-dependent kinase 1
CFL1	cofilin-1
CHMP	charged multivesicular body protein
CHP	calcineurin B homologous protein 1
CLTA	clathrin light chain A
CSF1R	macrophage colony-stimulating factor 1 receptor
DNM	dynamin
CTSB	cathepsin B;
DNM2	dynamin 2
DNM1L	dynamin-1-like protein
EDIL3	EGF-like repeat and discoidin I-like domain-containing protein 3
EHD	EH domain-containing protein
ENS	endocytosis
ER	endoplasmic reticulum
ESI	electrospray ion
EV71	enterovirus 71
EVP1	EV71 VP1 subunit
FCGR1	high affinity immunoglobulin gamma Fc receptor I
FDPS	farnesyl pyrophosphate synthase
FLNA	filamin-A
FLT1	vascular endothelial growth factor receptor 1
GM1	ganglioside M1
GSTO1	glutathione *S*-transferase ω-1
HERC4	probable E3 ubiquitin-protein ligase HERC4
HFMD	hand-foot-and-mouth disease
HIST1H1E	histone H1E
HSPA4	heat shock 70 kDa protein 4
IFI35	interferon-induced 35 kDa protein
IQSEC1	IQ motif and SEC7 domain-containing protein 1
ISRE	IFN-stimulated response element
iTRAQ	isobaric tags for relative and absolute quantitation
MS	mass spectrometry
JAK	Janus kinase
KLHL9	kelch-like protein 9
LAMP-1	Lysosome-associated membrane glycoprotein 1
LC-MS	liquid chromatography-tandem mass spectrometry
LT	heat-labile toxin
LTB	B subunits of LT
MAPK	mitogen-activated protein kinase
NFATC2	nuclear factor of activated T-cells, cytoplasmic
NFKB1	nuclear factor NF-κB p105 subunit
OAS1A	2′-5′-oligoadenylate synthase 1A
PANTHER	protein annotation through evolutionary relationship
PIAS4	E3 SUMO-protein ligase PIAS1
PPIL2	peptidyl-prolyl cis-trans isomerase-like 2
PPM1A	protein phosphatase 1A
PTPN7	tyrosine-protein phosphatase non-receptor type 7
Q-TOF-MS	source for quadrupole time-of-flight mass spectrometry
RAB5A	Ras-related protein Rab-5A
RAP1B	Ras-related protein Rap-1b
PRSS1	protease serine 1
ROS	reactive oxygen species
SLC11	solute carrier 11
SLFN	schlafen
SRC	proto-oncogene tyrosine-protein kinase
STAM2	signal transducing adapter molecule 2
STAT	signal transducers and activators of transcription
STAU2	double-stranded RNA-binding protein Staufen homolog 2
SWAP70	switch-associated protein 70
TCP1	*t*-complex protein 1
TGFB1	transforming growth factor β-1
UBE2R2	ubiquitin-conjugating enzyme E2 R2
UBMP	ubiquitin mediated proteolysis pathway
VPS	vacuolar protein sorting-associated protein
WWP2	NEDD4-like E3 ubiquitin-protein ligase WWP2
XIAP	E3 ubiquitin-protein ligase XIAP
ZFYVE9	Zinc finger FYVE domain-containing protein 9
