# Supplementary material for: Quantitative Proteomic Analysis of Escherichia coli Heat-Labile Toxin B Subunit (LTB) with Enterovirus 71 (EV71) Subunit VP1
Source: Int J Mol Sci. 2016 Aug 27;17(9):1419. doi: 10.3390/ijms17091419 (PMC5037698; doi:10.3390/ijms17091419)
Supplement: Supplementary file 1 [file ijms-17-01419-s001.pdf]

Supplementay Materials: Quantitative Proteomic Analysis of *Escherichia coli* Heat-Labile Toxin B Subunit (LTB) with Enterovirus 71 (EV71) Subunit VP1

Lin Liu, Yongping Ma, Huicong Zhou and Mingjun Wu

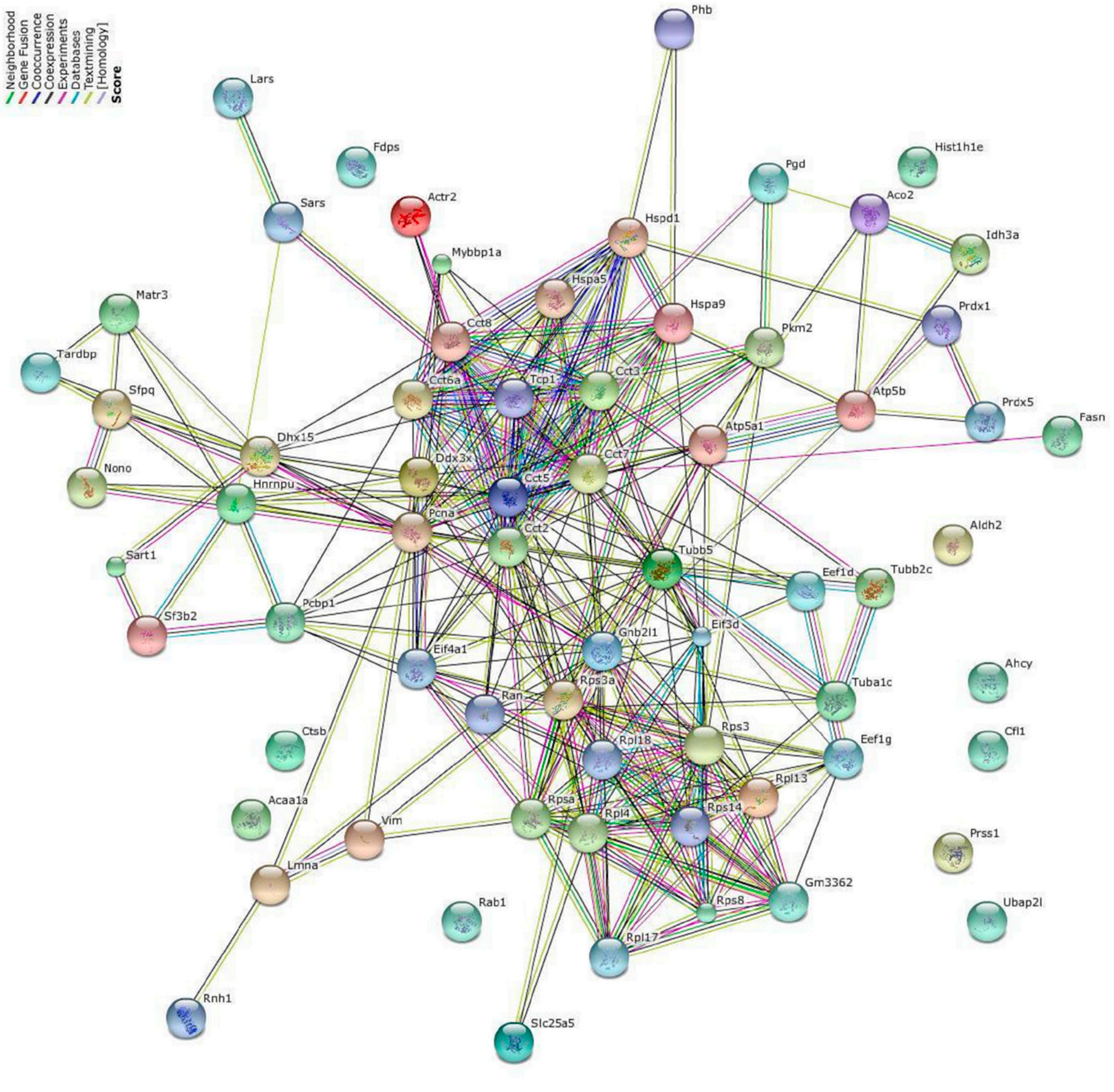

Figure S1. The prediction of protein-protein interactions pulled-down by LTB. The interactions map generated by STRING (confidence 0.4). Interaction proteins displayed from LTB pulled down proteins in mouse mø264.7.

Table S1. The LTB pull-down proteins.

Accession	Proteins	LTB	EVP1	LTB + EVP1
P52480	PKM	0.7311	0.1159	0.597
P19096	FASN	**1.6293**	0.8017	1.5417
D2KHZ9	GAPDH	0.2938	0.0982	0.5445
Q5FWJ3	VIM	0.3767	0.2399	0.9817
Q3U9G2	HSPA5	0.4365	0.1941	0.8872
P63038	HSPD1	0.3664	0.1343	0.929
P99024	TUBB5	1.4454	0.4613	1.556
P68372	TUBB4B	0.673	0.5861	0.8472
Q52L87	TUBA1C	**1.6749**	0.278	1.5996
P48678	LMNA	0.2466	0.2333	0.8166
Q03265	ATP5A1	0.2938	0.2355	0.6427
P38647	HSPA9	0.3802	0.177	0.7798
P56480	ATP5B	0.2489	0.4571	0.9376
Q3UM23	RNH1	1.0666	0.2582	0.9376
P11983	TCP1	0.8017	0.3802	1.0864
Q542X7	CCT2	0.8872	0.492	1.1482
Q99KI0	ACO2	0.5105	0.2582	0.8472
Q3U2W2	MYBBP1A	0.9727	0.9638	0.9727
Q3UJW1	ALDH2	0.2582	0.1225	0.5808
Q8VEK3	HNRNPU	0.7447	0.597	1.3804
P35700	PRDX1	0.1888	0.0738	0.4699
Q6A0F1	CCT8	1.0471	0.4875	1.1482
P80316	CCT5	0.5346	0.3342	1.1272
Q3TVS6	CTSB	0.177	0.4325	0.7516
P68040	GNB2L1	**1.5136**	0.6138	0.597
Q3U4U6	CCT3	0.787	0.4613	1.1376
Q5F2A7	EIF4A1	0.9908	0.9817	1
Q7TSZ3	LARS	0.9376	0.8954	1.5136
Q545A2	SLC25A5	0.2128	0.2992	0.6982
B9EKE9	DDX3X	**2.421**	0.6081	1.4454
Q3TI05	CCT6A	1.0965	0.3105	1.0965
Q3TET0	CCT7	0.8241	0.2443	1.0471
Q544Y7	CFL1	0.8241	0.0483	0.4875
Q9DCD0	PGD	1.1482	0.2208	0.6081
Q4FZK2	EEF1G	**1.9953**	0.6486	0.8241
P60335	PCBP1	0.8241	0.5916	1.0186
Q921H8	ACAA1A	1.4191	0.4613	1.0186
Q5BL18	MATR3	0.9462	0.9638	0.9638
Q8C483	SARS	0.8017	0.3221	0.2965
Q5SW83	ACTR2	0.6918	0.8017	0.9204
Q91ZH2	PCNA	0.7943	0.1472	1.2246
Q80X50	UBAP2L	0.871	0.5105	1.1169
P67778	PHB	0.6982	0.2831	0.912
Q3UKJ6	DHX15	0.673	0.4285	1
Q4FJN9	FDPS	0.2679	0.1542	0.7178
B2CY77	RPSA	**1.6293**	0.7516	0.7516
P62827	RAN	1.028	0.1486	0.7244

Table S1. *Cont.*

Accession	Proteins	LTB	EVP1	LTB + EVP1
Q8VIJ6	SFPQ	0.7178	0.7178	1.2359
O70194	EIF3D	0.9638	0.9638	1.0093
Q5YLW3	RPS3	0.7178	0.492	0.6026
Q3TF14	AHCY	0.6026	0.177	0.8166
Q3UJB0	SF3B2	0.3251	0.3162	0.8017
Q544R5	TARDBP	0.8166	1.0965	0.8166
Q3UB66	RAB1	0.6026	0.1009	0.6138
Q3TF40	NONO	0.4656	0.4875	1.0765
Q80T06	EEF1D	0.912	2.3768	0.9638
Q9D6R2	IDH3A	0.597	0.6026	0.879
Q9D1S3	RPS3A1	1.2023	0.5297	0.6194
P99029	PRDX5	0.3698	0.1722	0.5546
Q9Z315	SART1	0.6855	0.7379	1.1066
O70569	RPS14	0.7656	0.6368	0.4365
Q564E8	RPL4	**18.5353**	7.4473	1.9409
Q497E9	RPS8	**12.4738**	3.6308	1.2134
Q91YK6	RPL23A	**3.2211**	0.3873	0.7311
Q9D824-3	FIP1L1	0.929	0.929	0.9727
P47963	RPL13	**4.0179**	1.3552	0.6194
Q642K1	RPL18	**13.5519**	1.3552	2.1478
Q6PHZ1	RPL17	**4.6559**	1.2706	0.8091
Q9Z1R9	PRSS1	0.8954	0.157	1.0765
P43274	HIST1H1E	**12.8233**	1.0568	2.7797

The bold face data showed the significantly up regulated proteins treated by LTB comparing to EVP1 and LTB + EVP1 treatments.
